# Acoustic Emissions in Rock Deformation and Failure: New Insights from Q-Statistical Analysis

**DOI:** 10.3390/e25040701

**Published:** 2023-04-21

**Authors:** Sergio C. Vinciguerra, Annalisa Greco, Alessandro Pluchino, Andrea Rapisarda, Constantino Tsallis

**Affiliations:** 1 Department of Earth Sciences, University of Torino, 10125 Torino, Italy; sergiocarmelo.vinciguerra@unito.it; 2 Department of Civil Engineering and Architecture, University of Catania, 95125 Catania, Italy; annalisa.greco@unict.it; 3 Department of Physics and Astronomy “Ettore Majorana”, University of Catania, 95123 Catania, Italy; alessandro.pluchino@ct.infn.it (A.P.); andrea.rapisarda@ct.infn.it (A.R.); 4 INFN Sezione di Catania, 95123 Catania, Italy; 5 Complexity Science Hub Vienna, 1080 Vienna, Austria; 6 Centro Brasileiro de Pesquisas Fisicas and National Institute of Science and Technology for Complex Systems, Rio de Janeiro 22290-180, Brazil; 7 Santa Fe Institute, Santa Fe, NM 87501, USA

**Keywords:** Acoustic Emissions, rock deformations and failures, interoccurrence events, nonadditive entropies, nonextensive statistical mechanics

## Abstract

We propose a new statistical analysis of the Acoustic Emissions (AE) produced in a series of triaxial deformation experiments leading to fractures and failure of two different rocks, namely, Darley Dale Sandstone (DDS) and AG Granite (AG). By means of q-statistical formalism, we are able to characterize the pre-failure processes in both types of rocks. In particular, we study AE inter-event time and AE inter-event distance distributions. Both of them can be reproduced with q-exponential curves, showing universal features that are observed here for the first time and could be important in order to understand more in detail the dynamics of rock fractures.

## 1. Introduction and Motivations

The analysis of the Acoustic Emissions (AE) in materials subjected to experimental tests is a very important technique for understanding their damage accumulation and failure modes [1,2,3,4,5,6,7].

AE are high-frequency elastic waves due to micromechanical damage induced by the micro-cracking. These emissions, therefore, represent indicators of deformation and fracturing processes occurring within a tested specimen and allow us to monitor cracking formation, growth and propagation. The detection of the AE can therefore allow both to understand how the damage accumulates and develops within the material and to monitor the final rupture. This information could be very useful in geophysics for the interpretation of field scale seismic signals and the understanding of earthquake precursors. Furthermore, a deep insight into the cracking propagation in the considered rocks can also be very important in civil engineering for monitoring the integrity of masonry bridges and buildings and developing strategies for the safe design of tunnels.

From a seismological point of view AE obey, as earthquakes, the Gutenberg–Richter relationship between frequency and magnitude [8,9]. Rock fracture and earthquake rupture are processes obeying similar statistics for source dimensions over more than eight orders of magnitude. In order to link experimental detailed studies to geophysical signatures recorded at the field scale, controlled laboratory rock deformation experiments, equipped with dense micro-seismic arrays for AE detection, have become a routinely used tool [10,11,12]. The failure process and the pre-failure crack growth and coalescence can be monitored at the laboratory scale via AE, which, analogously to earthquake ruptures at the field scale, obey similar statistics and power-laws in time, space and magnitude [13,14,15]. Source mechanism evolution with various effective pressure can also be inferred from AE, allowing us to determine the effect of the increasing lithostatic pressure on the fracturing mechanisms and failure modes [16].

Moreover, in a civil engineering context, a deep knowledge of the behavior of the foundation soil is mandatory in order to correctly take into account soil–structure interaction. Reliable information on the fracturing mechanisms of surrounding rocks is also very important in the seismic design of tunnels [17,18]. Excavation methods, dimensions and design parameters of a tunnel strictly depend on the mechanical properties of the rocks along its alignment. The mechanical information regarding soil and rocks is obtained by means of appropriate tests performed either in situ or from samples. In the case of tunnels, the surrounding rocks are subjected to distributed pressures, which may vary in direction and intensity. When samples of rocks are extracted, it is, therefore, important to reproduce in three-dimensional (3D) experimental tests the effects of the distributed confinement pressures. A reliable 3D laboratory test on soils and rocks is the triaxial test which allows us to evaluate the effect of confinement pressures on the failure load of the specimens [19,20,21,22]. Moreover, in this field, the analysis of AE provides important information on the fracturing mechanisms and global resistance of the involved rocks.

In this paper, we present a new analysis of the AE recorded during conventional triaxial deformation tests at confining pressures up to 40 MPa [16]. In particular, our experimental setup allows recording AE generated during the loading by the crack initiation, propagation and growth, leading eventually to macro-fracture formation and sample failure. In this context, the use of non-extensive q-statistics has recently proved to be particularly effective in capturing some universal features which emerge during the crack’s propagation under loading [1,7]. In a previous article, some of the authors of this paper investigated, by means of q-statistics, AE in uniaxial compression experiments on samples of basalt and concrete subjected to cyclic loading [5]. The aim of the present study is to apply a similar statistical analysis to AE obtained in triaxial compression tests in order to take into account the effect of the confining pressure on the AE release and assess the influence of increasing confining pressure, different deformation and failure mode on the AE statistical properties. At variance with previous papers, not only have the time distributions been investigated but also a joint space-time analysis, which has enabled the quantification of several new phenomena.

## 2. Experimental Setup and Data Analysis

The AE used in this work have been recorded during conventional servo-controlled triaxial deformation experiments (Sanchez Technologies, Frépillon, France), installed at the University of Portsmouth, UK on cylindrical 40 mm × 100 mm samples with an array of twelve 1 MHz single-component Piezo-Electric Transducers for AE detection [23]. Further details on laboratory methods and experimental conditions can be found in [Benson et al., 2019]. Triaxial compression tests have been performed at 5, 10, 20 and 40 MPa on geologically and physically (i.e., fabric, porosity, grain size and cementation) different lithologies such as the Darley Dale Sandstone (DDS, porosity 14%) and Alzo Granite (AG, porosity < 1%). Further details on the AE data set and the lithologies can be found in [17]. The AE data sets have been analyzed in terms of source mechanisms time and spatial evolution before the rupture from some of the authors [17]. AE source mechanisms analysis has evidenced single fracture nucleation for AG and multiple fracture nucleation for DDS due to single or multiple competing dilatant and compactant regions. Fracture growth and propagation appear to be controlled by the different confinements, with increasing pressure controlling the time evolution and size of dilatant and compactant regions, eventually controlling crack coalescence into macroscopic fractures [16].

**Figure 1:**
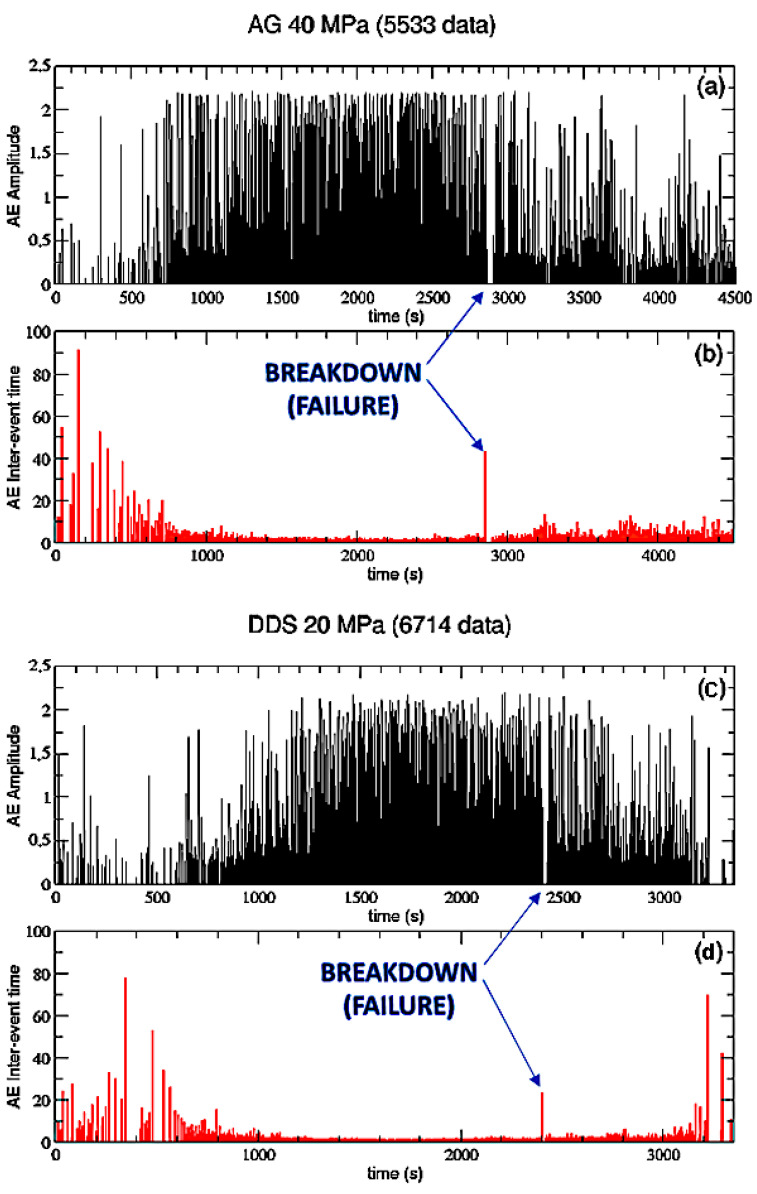
AE Amplitude and inter-event time as functions of time (s) for both AG 40 MPa, panels (**a**) and (**b**), and DDS 20 MPa, panels (**c**) and (**d**).

In order to give representative insights on the AE time and amplitude distribution prior to failure, we plot in Figure 1 the AE amplitude and the AE inter-event time as functions of time for two samples: AG at 40 MPa (panels (a) and (b), respectively) and DDS at 20 MPa (panels (c) and (d), respectively). The inter-event time δτ(n) is the time interval (in seconds) between two consecutive recordings AE(n) and AE(n−1) can be defined as:

$$\delta \tau \left(n\right)=t_{AE}\left(n\right)-t_{AE}\left(n-1\right)$$

where $t_{AE}\left(n\right)$ is the time at which the *n*-th AE event does occur and $t_{AE}\left(n-1\right)$ the time of the previous event.

The coupled analysis of AE amplitude and inter-event times reveals that AG shows an abrupt increase at the end of the elastic phase, between 500 and 1000 s, then AE amplitude remains quite constant until failure (which happens at about 2800 s); finally, it suddenly decreases during the stick-slip AE events occurring in the post-failure phase, with occasional AE clusters of higher amplitude driven by stress at the fault asperities. On the other hand, DDS presents a much more gradual AE amplitude increase from 500 to 1500 s, reaching steady values before failure (which happens at about 2400 s), then a decrease driven by the post-failure stick-slip processes. The difference between the two behaviors can be explained by the diverse deformation mechanisms acting on the lithologies. In particular, at the end of each sequence, a different macroscopic structure can be observed. For AG, a single damage cycle of crack nucleation and growth is sufficient to propagate fractures and develop the planar localization leading to dynamic failure. Whilst in DDS, it can take multiple cycles of nucleation for coalescence to take place due to interacting mechanisms induced by multiple fracture nucleation sites [16].

Thus, the DDS shows a much clearer premonitory phase, or foreshock, before a critical damage threshold which would allow coalescence into a larger-scale deformation structure. For both the samples, failure is characterized by a peak in the AE inter-event time, which suddenly appears after a sequence of very low values corresponding to the sequence of high amplitude events before rupture, corresponding to the transition from mm scale propagating microfractures to a fully developed cm scale fault zone.

In Table 1, we report, for each sample, the time *t_B_* at which the breakdown occurs, the total time *t_TOT_* of the experiment, the number N* of AE events before breakdown and the total number *N* of AE events present in the corresponding time series. In the following, we will investigate if *q*-statistics can help in revealing different structures in these data sets for increasing levels of confinement.

### 2.1. AE Amplitudes

To start, let us look at the amplitude of the probability density function (PDF) of the acoustic emissions for our eight considered samples. Firstly, we explore these distributions by dividing each time series into four parts (time intervals):

- From the beginning to 30% *t_B_*;
- From 30% *t_B_* to 70% *t_B_*;
- From 70% *t_B_* to breakdown;
- After breakdown.

Of course, the number of data included in each time interval could even be quite small; however, such a procedure could bring out otherwise hidden details of the process leading to the rupture.

**Figure 2:**
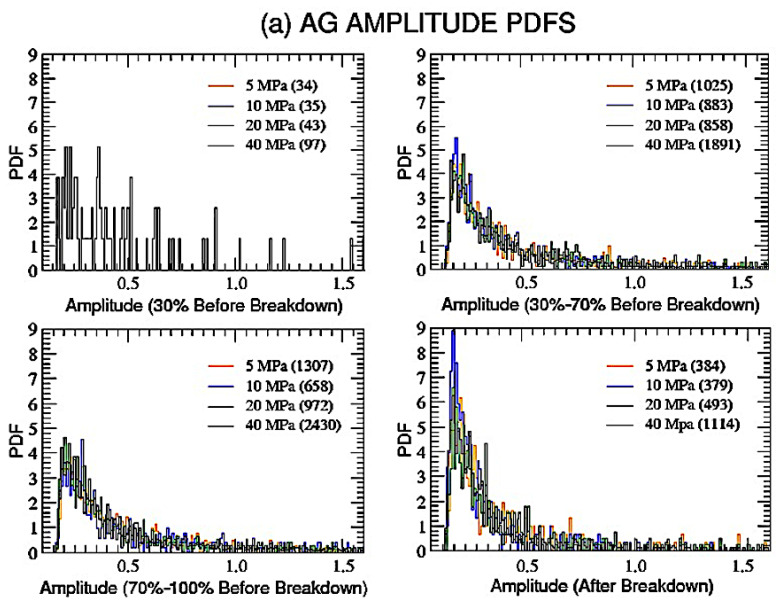

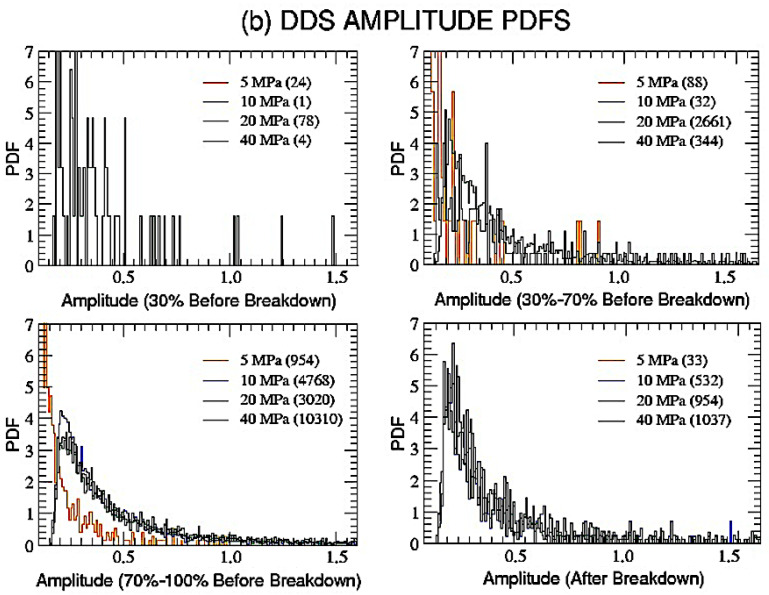
Amplitude probability distributions for both AG (**a**) and DDS (**b**) and for the four increasing levels of confinement. Each data set has been divided into four time intervals, three before breakdown (0–30%, 30–70%, 70–100%) and the last one after failure. In the legend, for each interval and each level of confinement, the number of AE events is reported in parentheses. Only distributions extracted from more than 50 events are shown in the panels.

In Figure 2, the amplitude PDFs for AG (a) and DDS (b) samples, within each time interval and for each level of confinement, are plotted; the number of events included in each time interval is also reported in legenda.

We will not show distributions corresponding to time intervals with less than 50 AE events, due, of course, to the too-poor statistics. We also anticipate that amplitude data for DDS at 5 MPa are not reliable since they have been affected by a problem with the pre-amplifier gain during the experiment.

Comparing the various panels for the two types of lithologies, one immediately notices several features which reveal some kind of universal behavior:

(i) Events in the first time interval (within 30% *t_B_*) are always too few to give consistent distributions, regardless of the material;
(ii) Distributions before and after failure are quite similar, again regardless of the material, with an initial sudden increase, a peak and a slow decrease for high amplitudes;
(iii) Distributions after failure are more peaked for both AG and DDS.

Our approach identifies the background general behavior driving the main mechanical phases of nucleation, growth, and coalescence of micro fractures in rock fracture, regardless of the prevalence of a specific phase in a specific deformation stage driven by the different lithologies and effective pressures.

**Figure 3:**
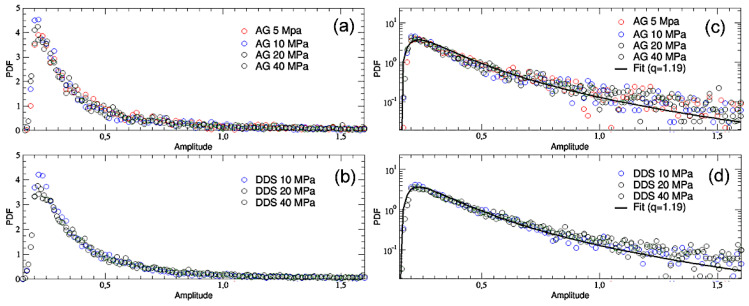
Amplitude probability distributions for the entire time series of AG (**a**–**c**) and DDS (**b**–**d**) at the four levels of confinement are reported, both in Lin-Lin (**a**,**b**) and in Log-Lin (**c**,**d**) scale. The Log-Lin curves have also been fitted with the non-linear function reported in Equation (1). Correlation coefficients of 0.68 for panel (**c**) and 0.74 for panel (**d**) confirm the good quality of the fits.

In Figure 3, the complete probability distributions for the whole time series of the different samples are reported both in Lin-Lin (Figure 3a,b) and in Log-Lin (Figure 3c,d) scale. It immediately appears that, for both the materials and for all the confinements, all the curves collapse one onto the others (with the exception of DDS 5 MPa, which has been excluded for the reasons explained before) and in all cases show power-law tails which overlap one over the others. The shape of the Lin-Lin curves is similar to that found in Figure 2, but the plots in Log-Lin scale tell us that we are in presence of fat tails. This result confirms that the amplitude of the AE events is scale-invariant and obeys a power-law as the earthquake’s frequency-magnitude distribution [9]. Both the distributions (Figure 3c) and (Figure 3d) can be well fitted (through a non-linear least squares method) with the following function, given by the product of a quadratic term and a *q*-exponential one (which, for *q* > 1, is a power-law):

(1) $$y=A_{0}{\left(x-x_{0}\right)}^{2}{\left(1-\left(1-q_{A}\right)\beta_{A}\left|x-x_{0}\right|\right)}^{\frac{1}{1-q_{A}}}$$

where $A_{0}$ = 7000, $\beta_{A}=40$, $q_{A}$ = 1.19 and $x_{0}=0.15$. The maximum of this distribution occurs precisely at $\left|x\right|=2/\left[\beta_{A}\left(3-2q_{A}\right)\right]$. Notice that the power-law, which is introduced as a pre-factor of the *q*-exponential function, is similar to the density of states which is present in the Planck law for the black-body radiation. Its origin is here possibly related to three-dimensional nearly isotropic stresses. Similar power-law pre-factors turn out to be necessary for diverse complex situations, such as the volume distributions in stock exchanges [24], distributions of transverse momenta of hadronic jets produced in proton-proton high-energy collisions at CERN/LHC [25] and COVID-19 peaks in the recent pandemic [26], among others.

### 2.2. AE Inter-Event Times 

Let us now shift our attention to the AE inter-event times. In particular, we will study the complementary cumulative (decumulative) probability distributions of AE inter-event times for both the AG and DDS samples, adopting only data before breakdown.

To build the decumulative distribution P (>δτ) of the inter-event time series, one has to report, for each value of δτ in the interval [0, 1000], the fraction of inter-event times which are greater than that value. Therefore, in the Log-Log scale, one could expect a curve starting from one for small values of δτ, then, after a certain inflection point, gradually decreasing with some kind of peculiar behavior for high values of δτ.

**Figure 4:**
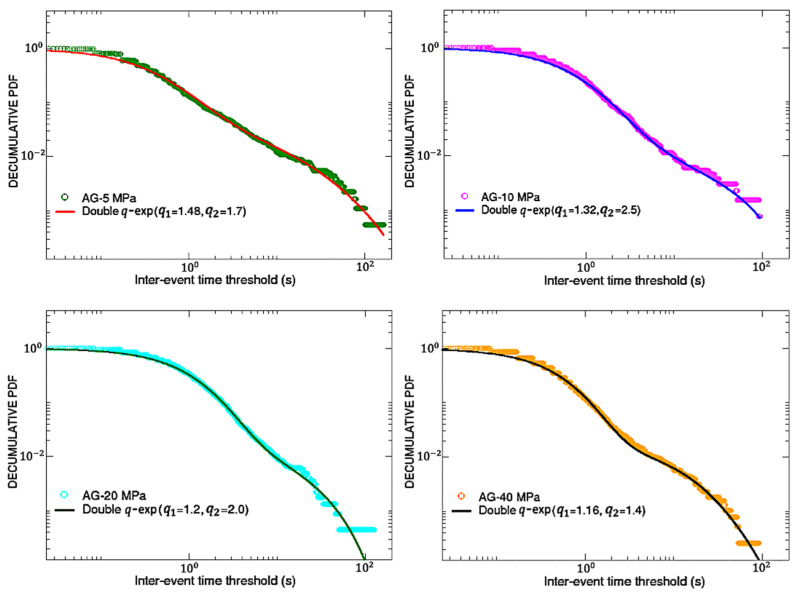
AG samples: double q-exp fits for the decumulative PDFs of inter-event times. All the correlation coefficients are above 0.8, thus confirming the good quality of the fits.

**Figure 5:**
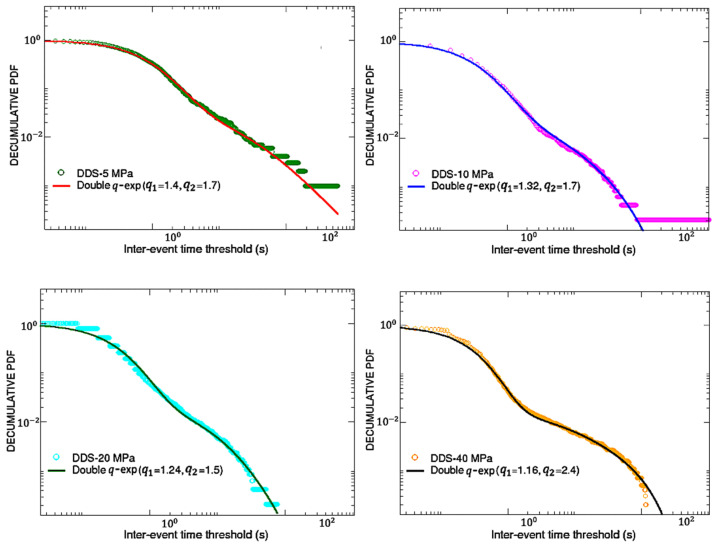
DDS samples: double q-exp fits for the decumulative PDFs of inter-event times. All the correlation coefficients are above 0.8, thus confirming the good quality of the fits.

In similar experiments [1,5], these decumulative distributions exhibited clear power-law tails, which can be framed in the context of the *q*-generalized thermo-statistics; actually, in these cases simple *q*-exponential functions were able to well fit the obtained curves, thus unveiling the fractal or multifractal nature of the breakdown process. In the results presented here, as shown in Figure 4 for AG and in Figure 5 for DDS, we found something more complex than the expected power-law tails. In fact, the decumulative PDFs for both types of analyzed materials seem to further change slope in correspondence of a second inflection point, whose time position decreases with increasing the confinement for DDS, while it seems to oscillate for AG. This new kind of behavior could still be described in the framework of *q*-thermo-statistics, but adopting the following more general fitting function [27]:

(2) $$y=A{\left(1-\left(1-q_{1}\right)\beta_{1}x\right)}^{\frac{1}{1-q_{1}}}+\left(1-A\right){\left(1-\frac{\lambda }{\beta_{2}}+\frac{\lambda }{\beta_{2}}e^{\left(q_{2}-1\right)\beta_{2}x}\right)}^{\frac{1}{1-q_{2}}}$$

It is composed of the sum of a first standard *q*-exponential function (with normalization factor 0<A<1, inverse temperature $\beta_{1}$ and entropic index $q_{1}$) and a second function containing (in addition to a normalization factor (1−A), an inverse temperature $\beta_{2}$ and an entropic index $q_{2}$) a further parameter λ>0, which ensures that the total function monotonically vanishes for increasing inter-times with appropriate behavior. See [28] for a similar crossover in the area-preserving dynamics of the standard map for intermediate values of the control parameter. It has been shown, for instance in [27,28,29,30], that the fitting parameter *A* is directly related to the comparative sizes, in the nontrivial phase space of the system, of the visitations of the regions related to q-exponentials characterized either by *q*_1_ or by *q*_2_.

Looking at Figure 4 and Figure 5, it can be appreciated that function (2) is able to well-fit (through a non-linear least squares method) all the PDFs, regardless of the material, provided that the following parameters are chosen.

In the previous table, Table 2, one can notice that, for any material and confinement, typically $\beta_{1}\gg \beta_{2}$ and $q_{1}<q_{2}$. Moreover, in correspondence of the same amount of confinement, values of A coincide for AG and DDS. A double *q*-exponential behavior, although rare, occasionally emerges in complex systems. Such is the case for the nucleotide inter-distances in DNA sequences of Homo Sapiens [31].

### 2.3. AE Positions and Inter-Event Distances

**Figure 6:**
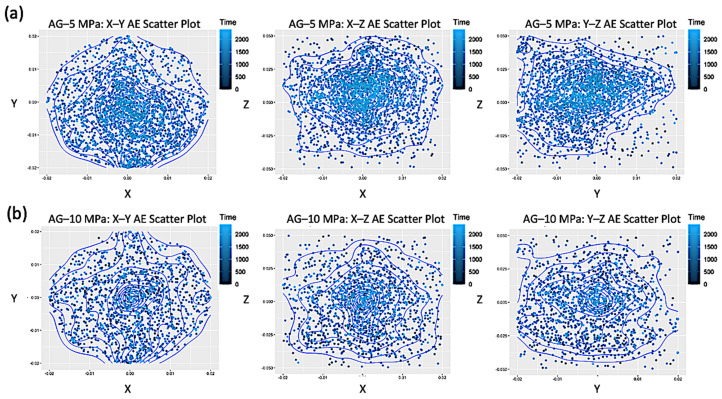

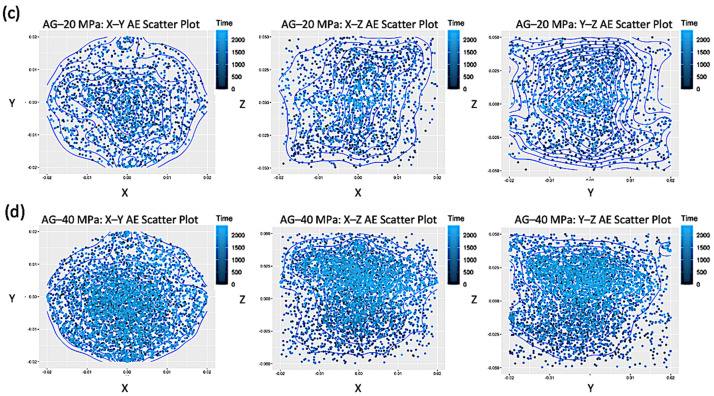
Scatter plots of the time-ordered positions before rupture (blue scale of decreasing intensity), projected on the three coordinate planes X–Y, X–Z and Y–Z, for AE events in the samples AG–5 MPa (**a**), AG–10 MPa (**b**), AG–20 MPa (**c**), AG–40 MPa (**d**).

**Figure 7:**
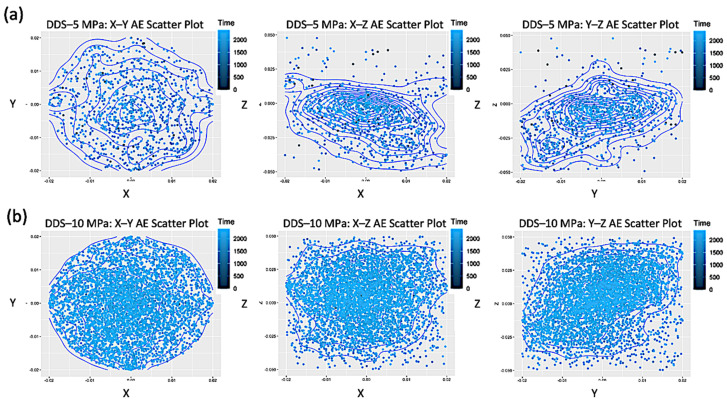

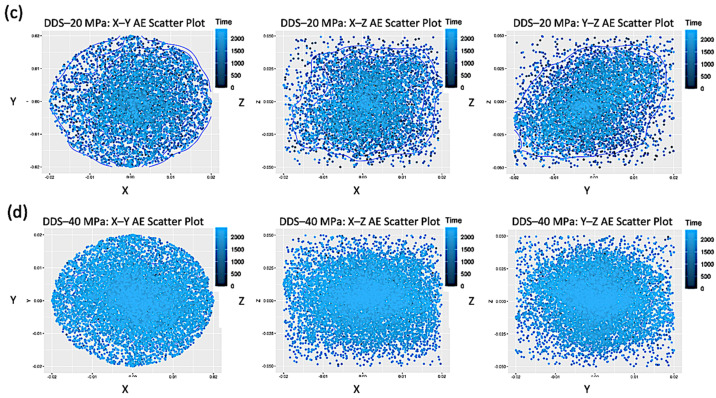
Scatter plots of the time-ordered positions before rupture (blue scale of decreasing intensity), projected on the three coordinate planes X–Y, X–Z and Y–Z, for AE events in the samples DDS–5 MPa (**a**), DDS–10 MPa (**b**), DDS–20 MPa (**c**), DDS–40 MPa (**d**).

Finally, let us investigate the eventual clustering in space of the AE events. In order to do this, we first explore the behavior of their subsequent positions (expressed in meters), ordered in time before breakdown (with a blue scale of decreasing intensity) and projected on the three planes X-Y, X-Z and Y-Z, for both AG (Figure 6) and DDS (Figure 7) samples with the usual confinements.

Spatial distributions of AE show a higher clustering of events in AG, where fracturing occurs throughout localized planar fractures, while more scattered nucleation centers, related to dilatant patches, occur before failure in DDS.

It is also useful to study the probability distribution of the AE inter-event distances (also expressed in meters), defined as the metric distance between the 3D spatial positions of two subsequent recorded AE events inside a given sample during an experiment:

(3) $$d\left(n\right)=\sqrt{{\left[x_{AE}\left(n\right)-x_{AE}\left(n-1\right)\right]}^{2}+{\left[y_{AE}\left(n\right)-y_{AE}\left(n-1\right)\right]}^{2}+{\left[z_{AE}\left(n\right)-z_{AE}\left(n-1\right)\right]}^{2}}$$

**Figure 8:**
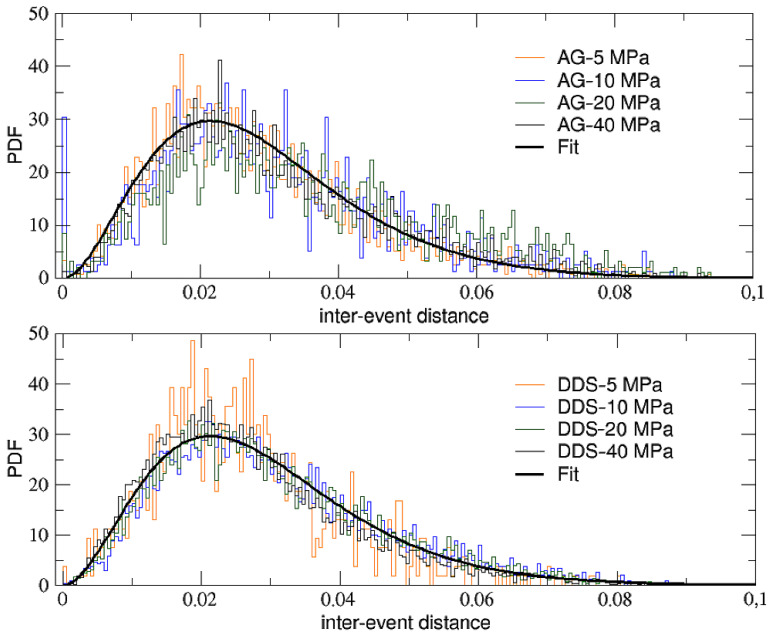
PDFs of the AE inter-event distance for AG (top panel) and DDS (bottom panel) at the various levels of confinement. Curves in both panels have been fitted with the function in Equation (4). Correlation coefficients of, respectively, 0.81 (top panel) and 0.87 (bottom panel), confirm the good quality of the fits.

In Figure 8, we plot the distributions of the inter-event distances before rupture obtained for both AG (top panel) and DDS (bottom panel) at the different levels of confinement. What we observe is a Planck-like distribution, with a maximum and an asymmetric tail, which can be well fitted (through a non-linear least squares method) by the following function:

(4) $$y=\frac{A\;x^{3}}{e^{Bx}-1}$$

where the values of the two fitting parameters (*A* = 4.8 × 10^7^ and *B* = 132) are independent of the type of materials and of the confinement, thus revealing again some kind of universal behavior. On the other hand, it is well visible a single narrow peak around zero for some AG samples, in particular those with intermediate levels of confinement, while this peak is completely absent for DDS samples. This different behavior might be explained by the single fracture nucleation mechanisms observed for AG, which implies high spatial clustering. For DDS samples, instead, multiple fracture nucleation mechanisms due to multiple fracturing regions have been observed [16] and these imply a diffused seismicity and a low clustering.

**Figure 9:**
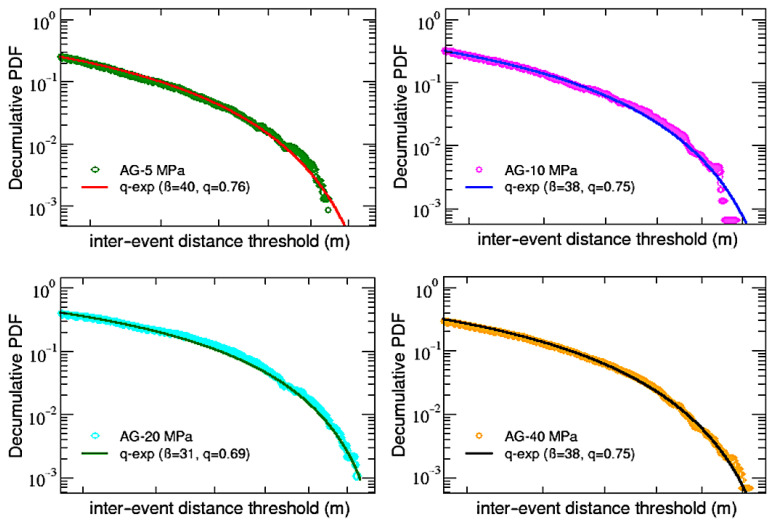
AG samples: single q-exp fits for the decumulative PDFs of inter-event distances.

**Figure 10:**
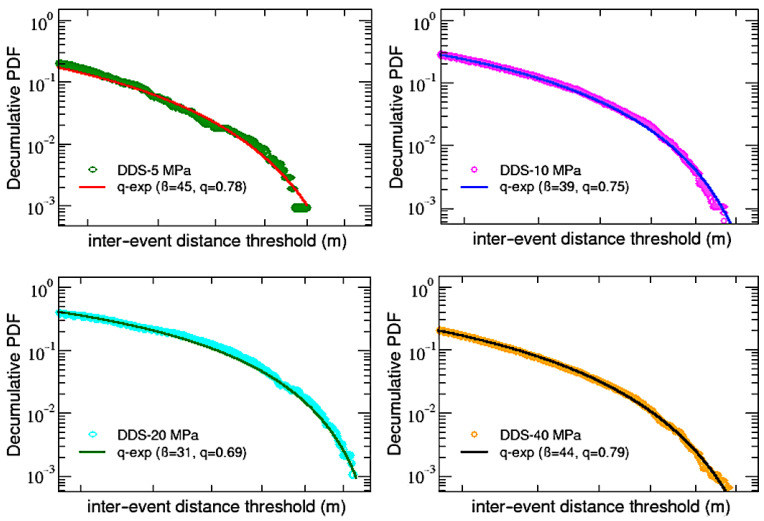
DDS samples: single q-exp fits for the decumulative PDFs of inter-event distances.

To close this analysis, let us finally look at the decumulative distributions P (>d) of the inter-event distances before rupture, which are shown in Figure 9 for AG samples and in Figure 10 for DDS ones. 

As for the inter-event times’ decumulative PDFs, in this case, it is also possible to fit (through a non-linear least squares method) all the distributions with *q*-exponential functions, but in this case, it is enough to use single standard *q*-exponentials with inverse temperature β and an entropic index *q*. Notice that, in analogy to what has been found in [32], values of the entropic indexes are all below 1, not indicating a power-law behavior but revealing the presence of a cut-off in the distributions. Moreover, as shown in Table 3, in agreement with a further finding of [32], calling $q_{\tau }$ the first entropic index $q_{1}$ obtained for the inter-event time PDFs (see Table 2) and $q_{d}$ the entropic index just found for the inter-event distance, it can be noticed that the sum $q_{\tau }+q_{d}$ oscillates around 2 for all the samples, regardless of both material and confinement. This result is also similar to what has been observed in regional seismicity data from Japan and California and numerically verified using the two-dimensional Burridge–Knoppoff model [32].

**Figure 11:**
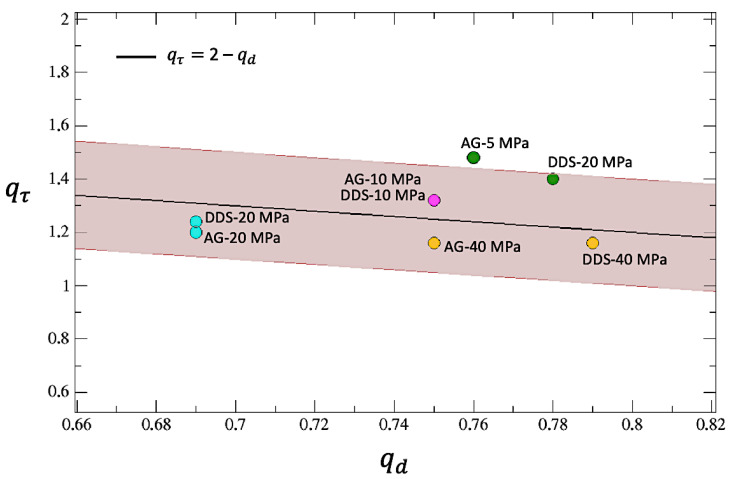
The entropic index $q_{\tau }$ is reported as a function of $q_{d}$ for the eight considered samples (colored circles) and it is compared with the line $q_{\tau }=2-q_{d}$. The ±10% area around the line is colored in brown. Notice that the points corresponding to AG-10 MPa and DDS-10 MPa coincide.

In Figure 11, the entropic index $q_{\tau }$ is reported as a function of $q_{d}$. The figure clearly shows that the linear behavior holds quite well with an error of 10% for almost all the samples.

As rock fracture and earthquake rupture are processes obeying similar statistics for source dimensions over more than eight orders of magnitude [9] the results of this study can be meaningful, within the scaling limitations, to larger scale mechanisms, such as the collapse prediction of building materials and the interpretation of deformation mechanisms preceding and accompanying earthquake ruptures. Our findings at the laboratory scale reveal that regardless of the deformation mechanisms taking place at different effective pressures and for different lithologies, the macroscopic coalescence into a larger-scale deformation structure and its seismic output would be controlled by the multiscale transition from mm scale fractures into a cm scale fault zone. Within the limitations of upscaling, the micromechanisms observed can have relevance for understanding the deformation mechanisms observed at the field scale, providing new insights for developing monitoring strategies for earthquake precursory detection.

## 3. Conclusions

We investigated by means of q-formalism the Acoustic Emissions (AE) produced in a series of triaxial deformation experiments leading to fractures and failure of two different materials, namely, Darley Dale Sandstone (DDS) and AG Granite (AG). We have shown that pre-failure processes in both types of rocks, and in particular AE inter-event time and AE inter-event distance distributions, can be reproduced with q-exponential curves, showing universal features that have been observed here for the first time. This quantitative characterization could be important in order to understand more in detail the processes of rock fracture dynamics and deformation mechanisms. It is certainly a step forward and very useful to study more in-depth how the obtained results, which give support to similar previous investigations where q-statistics has been used, may have important applications in civil engineering and in the analysis of soil–structure interaction, as well as in geophysics for the comprehension of seismic signals preceding and accompanying earthquakes.

## Figures and Tables

**Table 1:** Details of the time series for the different samples.

Sample	*t_B_*	*t_TOT_*	N* Events before Breakdown	Tot. *N* Events
AG-5 MPa	2398 s	3395 s	2367	2751
AG-10 MPa	1767 s	2445 s	1577	1956
AG-20 MPa	2160 s	2973 s	1874	2367
AG-40 MPa	2857 s	4566 s	4419	5533
DDS-5 MPa	2400 s	2540 s	1067	1100
DDS-10 MPa	5808 s	6248 s	4802	5334
DDS-20 MPa	2409 s	3348 s	5760	6714
DDS-40 MPa	9710 s	11,736 s	10,659	11,696

**Table 2:** Details of the fitting parameters of Equation (2) for the eight samples considered.

Sample	A	$\beta_{1}$	$q_{1}$	$\beta_{2}$	$q_{2}$	λ
AG-5 MPa	0.975	3.5	1.48	0.01	1.7	0.09
AG-10 MPa	0.98	2.0	1.32	0.02	2.5	0.19
AG-20 MPa	0.98	1.3	1.2	0.04	2.0	0.13
AG-40 MPa	0.983	2.6	1.16	0.017	1.4	0.12
DDS-5 MPa	0.975	1.5	1.4	0.0001	1.7	0.055
DDS-10 MPa	0.98	4.0	1.32	0.02	1.7	0.19
DDS-20 MPa	0.98	4.1	1.24	0.017	1.5	0.20
DDS-40 MPa	0.983	4.6	1.16	0.015	2.4	0.19

**Table 3:** The sum of the entropic indices $q_{\tau }$ and $q_{d}$ for the 8 considered samples oscillates around 2.

Sample	$q_{\tau }$	$q_{d}$	$q_{\tau }+q_{d}$
AG-5 MPa	1.48	0.76	2.24
AG-10 MPa	1.32	0.75	2.07
AG-20 MPa	1.2	0.69	1.89
AG-40 MPa	1.16	0.75	1.91
DDS-5 MPa	1.4	0.78	2.18
DDS-10 MPa	1.32	0.75	2.07
DDS-20 MPa	1.24	0.69	1.93
DDS-40 MPa	1.16	0.79	1.95

## Data Availability

Data are available under request.

## Abbreviations

AE	Acoustic Emissions
DDS	Darley Dale Sandstone
AG	Alzo Granite
3D	Three dimensional
PDF	Probability density function
Lin-Lin	Linear-Linear
Log-Lin	Logarithmic-Linear
Log-Log	Logarithmic-Logarithmic
